# Guided Inhalation via Electronic Monitoring in Children With Uncontrolled Asthma (the IMAGINE Study): Randomized Controlled Trial

**DOI:** 10.2196/78526

**Published:** 2025-11-14

**Authors:** Esther Sportel, Kris Movig, Bernard Thio, Mattienne Van der Kamp, Job Van der Palen, Marjolein Brusse-Keizer

**Affiliations:** 1 Department of Clinical Pharmacy Medisch Spectrum Twente Enschede The Netherlands; 2 Department of Pediatrics Medisch Spectrum Twente Enschede The Netherlands; 3 Biomedical Signals and System Department University of Twente Enschede The Netherlands; 4 Department of Epidemiology Medisch Spectrum Twente Enschede The Netherlands; 5 Section Cognition, Data and Education University of Twente Enschede The Netherlands; 6 Health Technology and Services Research University of Twente Enschede The Netherlands

**Keywords:** clinical outcomes, inhalation technique, adherence, smart inhaler, pediatrics, eHealth, asthma

## Abstract

**Background:**

Pediatric asthma is the most common chronic illness among children in the Netherlands. Scheduled hospital visits provide limited insight into therapy adherence and inhalation technique, which are critical for disease control. Smart inhalers that provide immediate feedback may offer a solution for monitoring and improving these parameters at home, leading to better asthma control.

**Objective:**

This study aimed to improve asthma control through immediate feedback on therapy adherence and inhalation technique, with the use of a smart inhaler.

**Methods:**

The IMAGINE (Improving Adherence by Guiding Inhalation via Electronic Monitoring) study was a randomized controlled trial consisting of 3 phases: an observational run-in phase in which adherence and technique were recorded, a randomized phase with feedback for the intervention group and recording for the control group, followed by an observational phase with only recording for both groups. Asthma control was measured with clinical outcomes including predicted forced expiratory volume in 1 second, lung function reversibility, lung function variability, the Asthma Control Test, and the Childhood Asthma Control Test.

**Results:**

Between October 2019 and October 2023, a total of 34 children were enrolled and randomized. Overall, improvements were observed at the end of phase 2 in clinical parameters (reversibility, lung function variability, Asthma Control Test, and Childhood Asthma Control Test), except for predicted forced expiratory volume in 1 second. However, no significant differences between the intervention and control groups over time were observed. At the end of phase 2, 87% (13/15) of control participants and 78% (10/13) of intervention participants met one or more predefined clinical criteria. Inhalation technique and therapy adherence did not differ over time between the groups (*P*=.70 and *P*=.14, respectively).

**Conclusions:**

While smart inhaler feedback did not lead to better outcomes compared with no feedback, clinical improvements were observed in both groups. Future studies should explore how adaptive smart inhaler interventions can be optimized to support personalized care and enhance patient ownership in asthma management.

**Trial Registration:**

International Clinical Trials Registry Platform NL-OMON50093; https://tinyurl.com/yws24rxy

**International Registered Report Identifier (IRRID):**

RR2-10.1186/s13063-020-04694-4

## Introduction

Pediatric asthma is the most prevalent chronic disease among children in the Netherlands, affecting between 5% and 10% of children up to 12 years of age [1]. Asthma is defined as a chronic inflammatory disorder of the airways resulting in airflow limitation and is characterized by bronchial hyperresponsiveness to a variety of stimuli [2]. The initial management of asthma is guided by the activity of the disease [3,4], requiring ongoing monitoring of asthma control to determine whether current therapies should be maintained or adjusted [3,5,6]. Accordingly, international guidelines [3,7] recommend that physicians reassess therapy adherence, the quality of inhalation technique, environmental factors, and associated comorbidities before considering escalation of pediatric asthma therapy [5,6,8,9].

The two prevalent factors leading to asthma control failure in children are poor therapy adherence and an inadequate inhalation technique, as evidenced by multiple studies [9-12]. Approximately 50% of children exhibit poor adherence to asthma medication, a factor linked to an increased frequency of exacerbations [13-17]. Good adherence has been significantly associated with improved asthma control [18]. Additionally, a substantial proportion of incorrect inhaler handling has been reported among children with asthma, with appropriate use remaining low, which may lead to incomplete drug delivery and increased asthma morbidity [19].

Given that therapy adherence and the inhalation technique followed at home cannot be reliably assessed during a hospital visit [20], home-based monitoring using smart inhalers offers a promising solution [21]. A randomized controlled trial demonstrated that electronic adherence monitoring with daily reminder alarms can enhance adherence, reduce the need for oral steroids, and decrease hospital admissions [22]. Other systematic reviews have emphasized that education combined with repeated feedback on inhaler technique is critical for improving proper device use [19,23].

In this context, AMIKO developed the Respiro device [24], an innovative add-on for conventional inhalers that measures both therapy adherence and inhalation technique by recording vibration patterns. The device captures key parameters including the time of use per inhalation, dosing intervals, peak inspiratory flow, inhalation duration, and the orientation of the device during medication administration. Connected to a patient app, Respiro delivers immediate smart feedback on adherence and technique. The first randomized controlled trial using this device, known as Improving Adherence by Guiding Inhalation via Electronic Monitoring (IMAGINE), was designed to evaluate its impact on asthma control in children [25,26].

The primary hypothesis of this study was that participants assigned to the feedback group would more often demonstrate clinical improvement—defined by 7 pediatric clinical criteria related to predicted forced expiratory volume in 1 second (predFEV_1_), lung function reversibility, lung function variability (LFV), and Childhood Asthma Control Test (C-ACT)—compared with participants assigned to the control (no feedback) group. Secondary aims included the assessment of therapy adherence and inhalation technique across both study groups.

## Methods

### Study Design

**Figure 1:**
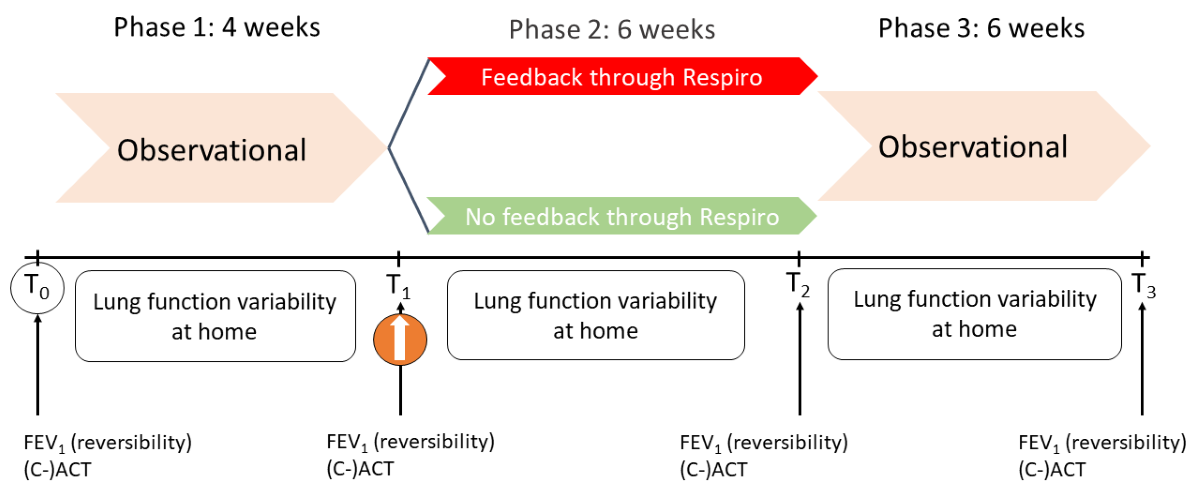
Study design and outcome measurements. The measurements were done at 4 points: T0, the start of the study; T1, the start of phase 2; T2, the start of phase 3; and T3, the end of the study. At T1, the participants were randomized. C-ACT: Childhood Asthma Control Test; FEV1: forced expiratory volume in 1 second.

The IMAGINE study was a 3-phase randomized controlled interventional study (Figure 1). For more detailed information, see the published protocol by Sportel et al [27]. The CONSORT-EHEALTH (Consolidated Standards of Reporting Trials of Electronic and Mobile Health Applications and Online Telehealth) guideline (version 1.6.1) was followed (Multimedia Appendix 1) [28]. Recruitment took place from October 2019 through October 2023.

Phase 1 was a 4-week observational period in which adherence and technique were measured by the Respiro device, without providing any feedback to children. At T1, randomization to feedback or no feedback took place (1:1). The randomization at T1 was a 1:1 blocked randomization, performed by an independent researcher. In addition, the randomization was stratified by age (≥12 years vs <12 years) and the use of nasal corticosteroids (prescribed vs nonprescribed). Patients in the intervention group received feedback from their smart device, while patients in the control group received no feedback. This interventional phase (phase 2) lasted 6 weeks. Blinding was not possible for either the participants or the staff due to the nature of this study. Phase 3 consisted of a 6-week observational follow-up during which adherence and technique were measured by the Respiro device but none of the patients received feedback. Treating physicians did not have access to adherence or inhalation technique data at T1 or T2, and no feedback based on these data was provided to patients.

### Ethical Considerations

The protocol was approved by the medical ethics committee of the University of Twente (P19-09), registered in Clinical Trials Registry Platform (trial number NL-OMON50093), was conducted in accordance with the Declaration of Helsinki, and was consistent with International Council for Harmonization of Technical Requirements for Pharmaceuticals for Human Use and Good Clinical Practice and the applicable regulatory requirements. Written consent was obtained from all children and their parents. In case participants were younger than 12 years, both parents or guardians signed the informed consent, for participants aged between 12 and 16 years both the child and the parents or guardians signed the informed consent, and for participants older than 16 years, the child was allowed to sign independently. The ethics committee approved the informed consent procedure, including the provision that participants aged 16 years and older could provide written consent independently, without requiring parental or legal guardian consent.

All research activities were conducted in accordance with ethical standards, including strict adherence to the principles of privacy and confidentiality to safeguard participants’ data and identities. No compensation was offered for participation in the study.

### Recruitment

All participants were aged between 6 and 18 years, had uncontrolled moderate to severe asthma, and were outpatients at a large teaching hospital in the Netherlands. Asthma was considered uncontrolled when the C-ACT score was below 20 or lung function reversibility on short-acting bronchodilator of at least 12% [6]. The diagnosis of moderate or severe asthma was made by the same pediatric pulmonologist for every child based on recommendations in the Global Initiative for Asthma (GINA) guidelines [29].

The Respiro device had to be compatible with the controller medication of the participants, which was possible for the Nexthaler (Chiesi Farmaceutici SpA). Switching from a metered dose inhaler to a dry powder inhaler was allowed, as long as the medication remained the same. Participants were instructed in the use of the new inhaler before entering the study and were required to use this device for at least 1 month before inclusion. If children, or their parents (if aged <12 years), were unable to speak or understand the Dutch language, they were excluded from participating in this study. The same applied to children with other chronic conditions that could potentially influence lung function.

### The Respiro Add-On Device

The Respiro add-on device is an attachment to the patient’s controller medication inhaler. This add-on device automatically tracks the generated flows of the user through the inhaler. The device uses an accelerometer that measures acceleration forces to determine orientation relative to gravity. It analyzes vibration patterns associated with inhaler use, which allows the identification of critical inhalation errors, such as failing to reach a sufficient peak flow, insufficient inhalation duration, and incorrect orientation of the inhaler. Respiro is connected via Bluetooth to an app on the patient’s smartphone, named the Respiro Mobile (Amiko Digital Health) app, and can be used for synchronizing the Respiro device. No major content changes were made to the system during the study.

### Intervention

**Figure 2:**
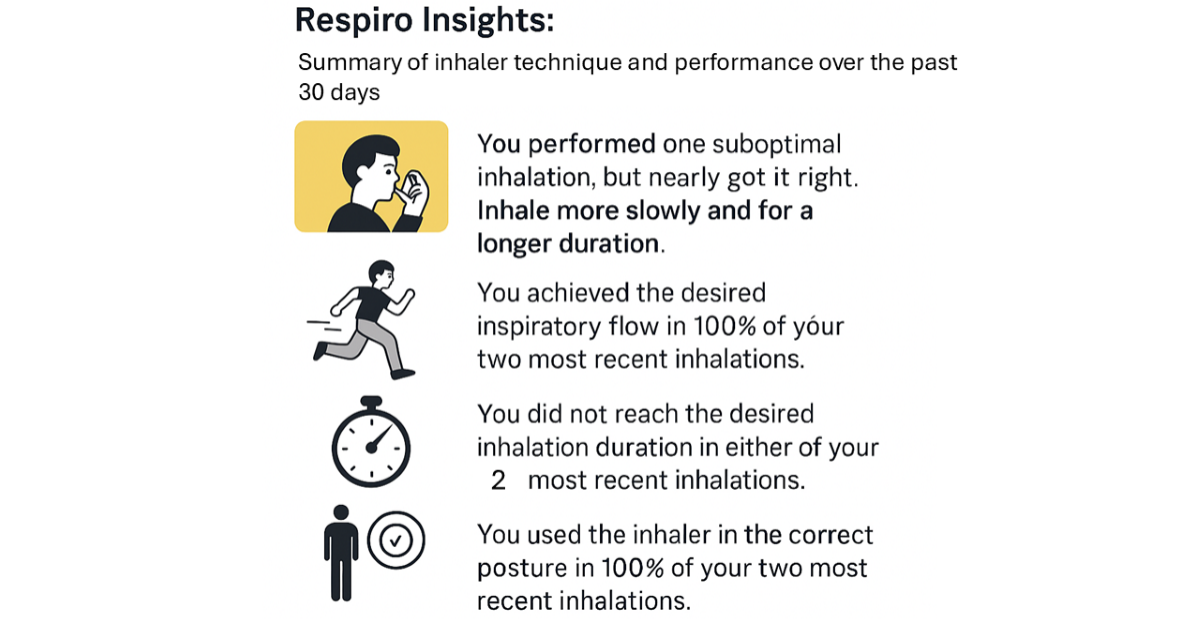
Examples of Respiro insights, motivational feedback messages, and visuals.

Participants in the intervention group received the Respiro Mobile app, which reminded them when it was time to inhale a dose. The app also provided personalized feedback to help participants optimize their inhalation technique. The feedback included motivational push messages immediately after each inhalation and provided feedback on inhalation quality trends with visuals and text (see Figure 2 for examples of messages, translated into English). The control group received an adapted Respiro Mobile app without reminders or feedback on the inhalation technique; it only registered inhalation use and technique observationally.

### Measurements

The children visited the hospital before the study began and after every phase. During the first appointment, the C-ACT and FEV_1_ reversibility tests were performed by a trained researcher to check if the children were eligible for the study. After being included, the following parameters were measured at fixed time points throughout the study duration (Figure 1). Baseline lung function reversibility and C-ACT were measured at the start of phase 1 to determine asthma control, and at the end of each phase. Lung function reversibility was determined by performing spirometry before and after administration of a short-acting beta-agonist and calculated using equation 1 (Multimedia Appendix 2). The C-ACT is a self-report, internationally accepted questionnaire assessing the patient’s perception of asthma complaints and asthma control for the previous 4 weeks. The regular Asthma Control Test was used for children 12 years and older and consisted of 5 questions. The C-ACT was used for children aged below 12 years and consisted of 4 questions for the child and 3 additional questions for the parents.

All participants received a handheld NuvoAir spirometer (Air Next, NuvoAir) [30] to perform spirometry at home. This device is connected via Bluetooth with the Air MD app (NuvoAir) on a mobile phone of the participant to register the spirometry results. The participants were trained and instructed to perform spirometry twice a week at home for the entire duration of the study. If deviations from the protocol were noticed regarding adherence to the twice-weekly home spirometry, the researcher actively contacted participants to encourage and maintain adherence as much as possible. In addition, each participant attended 4 scheduled study visits at the hospital, during which spirometry was performed together with the researcher. This ensured that reliable measurement points were consistently obtained throughout the study. After each phase, LFV was determined using the minimum and maximum value of the home FEV_1_ measurements of a specific participant during a particular phase using equation 2 (Multimedia Appendix 2).

### Asthma Control

The primary outcome of the IMAGINE study was the degree of asthma control, measured at the end of phase 2 [27]. Asthma control was evaluated based on 4 clinical parameters: FEV_1_, C-ACT, lung function reversibility, and LFV. Additionally, 7 predefined criteria were used to specifically assess pediatric asthma control. Clinical improvement in asthma control was defined as the fulfillment of one or more of these criteria. Criteria 3 and 5 were excluded from the primary outcome if they had already been met at baseline. An overview of these criteria and the corresponding categories for clinical improvement is provided in Multimedia Appendix 3.

The 7 criteria were as follows [6,31]:

1. A relative FEV_1_ increase of ≥10% at the end of phase 2, compared with the baseline value measured at the start of phase 1
2. An absolute C-ACT score increase of ≥3 points at the end of phase 2, compared with the baseline C-ACT score measured at the start of phase 1
3. A C-ACT score of ≥20 at the end of phase 2
4. A relative lung function reversibility decrease of ≥9% at the end of phase 2, compared with the baseline lung function reversibility measured at the start of phase 1
5. A lung function reversibility of <12% after administration of salbutamol at the end of phase 2
6. A relative LFV decrease of ≥10% during phase 2, compared with the LFV of phase 1
7. An LFV of ≤15% measured during the entire phase 2

### Therapy Adherence and Inhalation Technique

Therapy adherence and inhalation technique were considered secondary outcome measures. Therapy adherence was assessed as the actual medication intake during a specific phase divided by the prescribed medication intake per day, multiplied by the number of days during that phase (equation 3 in Multimedia Appendix 2). In addition, the percentage of adherence was qualified as insufficient (<75%) or good (≥75%).

The quality of the inhalation technique was assessed using two critical errors: (1) the peak inspiratory flow of less than 30 L/min [32] and (2) the inhalation duration was less than 1 second [33]. An inhalation was classified as incorrect when at least one of these 2 critical errors occurred. The overall inhalation technique was calculated as the percentage of correct inhalations (not containing a critical error) divided by the total number of inhalations during a specific phase (equation 4 in Multimedia Appendix 2). Inhalation technique was qualified as poor when ≥25% of the inhalations contained a critical error.

### Statistical Analysis

Baseline characteristics were presented as means with SD or medians with IQR for continuous variables depending on the distribution of the variable; categorical variables were presented as numbers with corresponding percentages. First, baseline characteristics were compared between the 2 groups. Continuous variables were tested with the independent 2-tailed *t* test or Wilcoxon rank-sum test, depending on distribution, and categorical variables were tested with a chi-square or Fisher exact test as appropriate.

Second, the mean changes over time for the continuous variables (the predFEV_1_, C-ACT scores, lung function reversibility, and LFV) were assessed using mixed model repeated measures analysis with fixed effects. A mixed model was created for each of these variables using the optimal covariance structure. Baseline values were subtracted from the follow-up values to obtain normally distributed values. The main parameter of interest was not only the group-by-time interaction but also the time and group effect that was assessed. All participants enrolled at baseline were included in the analyses.

Third, between-group analyses were performed to assess differences in performance for each of the 7 criteria by the Wilcoxon rank-sum test. Percentages of participants reaching the thresholds for improvement per criterion were compared with Fisher exact test.

Percentages of therapy adherence and critical inhalation errors were also assessed with a mixed model repeated measures analysis. For this, the mean percentage of therapy adherence and the mean percentage of inhalations containing critical errors for each participant in every phase were calculated. Percentages of adherence and critical errors during phase 1 were subtracted from the values of the following phases to obtain normally distributed values. In addition, a between-group comparison of participants achieving good therapy adherence and a proper inhalation technique in phases 1 and 2 was performed using the Fisher exact test.

As detailed in our published protocol (Sportel et al [27]), the sample size was calculated based on a 2-sample comparison of independent proportions (2-sided *Z* test), assuming improvement in asthma control in 10% of controls versus 40% of the intervention group, with 80% power; calculations were performed in PASS 11 (NCSS LLC). An interim analysis at 50% information was planned using O’Brien-Fleming spending (target final α=.049), with stopping for futility if the observed difference was ≤10% and early stopping for efficacy if *P*<.005; participants already enrolled completed all study phases. The resulting minimum required sample was 62, inflated by 10% for anticipated attrition to a recruitment target of 68 participants.

All these analyses were performed according to the intention-to-treat principle. The data were analyzed using SPSS (IBM Corp). *P*<.05 was considered statistically significant.

## Results

### Overview

**Figure 3:**
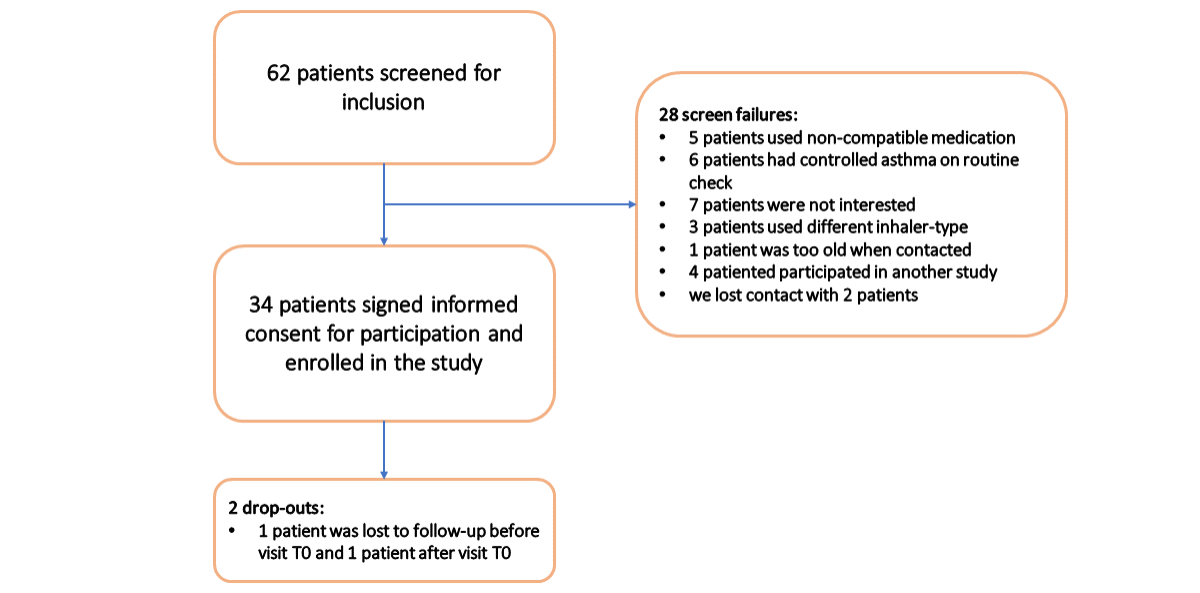
Participant flow graph.

Between October 2019 and October 2023, a total of 62 children with presumed uncontrolled asthma were approached to participate in the study, of whom 34 (55%) children were included and randomized (Figure 3). At T0, the children had a mean age of 11.7 (SD 2.7) years, 24 (71%) were male children, and the median C-ACT score was 18 (IQR 15-21). All demographic and baseline characteristics are presented in Table 1. These characteristics did not differ significantly between the control and intervention groups (Table 1). No significant difference in lung function reversibility at baseline was observed between the groups.

**Table 1:** Demographic characteristics and measurements at T0 of the 33 study participants.

Demographic characteristics and baseline measures	Control group (n=15)	Intervention group (n=18)	*P* value
Age (years), mean (SD)	11.1 (2.6)	12.2 (2.7)	.28
Height (cm), mean (SD)	147.7 (13.6)	156.9 (18.8)	.12
Gender (male), n (%)	13 (87)	10 (56)	.13
Nasal corticosteroid use, n (%)	11 (73)	10 (56)	.28
C-ACT^a^ score, median (IQR)	17.0 (16.0-19.0)	20.0 (10.8-22.3)	.48
C-ACT score >20, n (%)	2 (13)	9 (50)	.06
Predicted FEV_1_^b^ pre-SABA^c^ (%), median (IQR)	83.55 (73.0-94.3)	89.5 (70.0-103.5)	.45
FEV_1_ reversibility (%), median (IQR)	13.5 (8.8-24.3)	13.5 (4.0-19.0)	.54
FEV_1_ reversibility ≤12, n (%)	5 (33)	5 (28)	>.99

^a^C-ACT: Childhood Asthma Control Test.

^b^FEV_1_: forced expiratory volume in 1 second.

^c^SABA: short-acting beta-agonist.

### Clinical Parameters of Asthma Control

**Figure 4:**
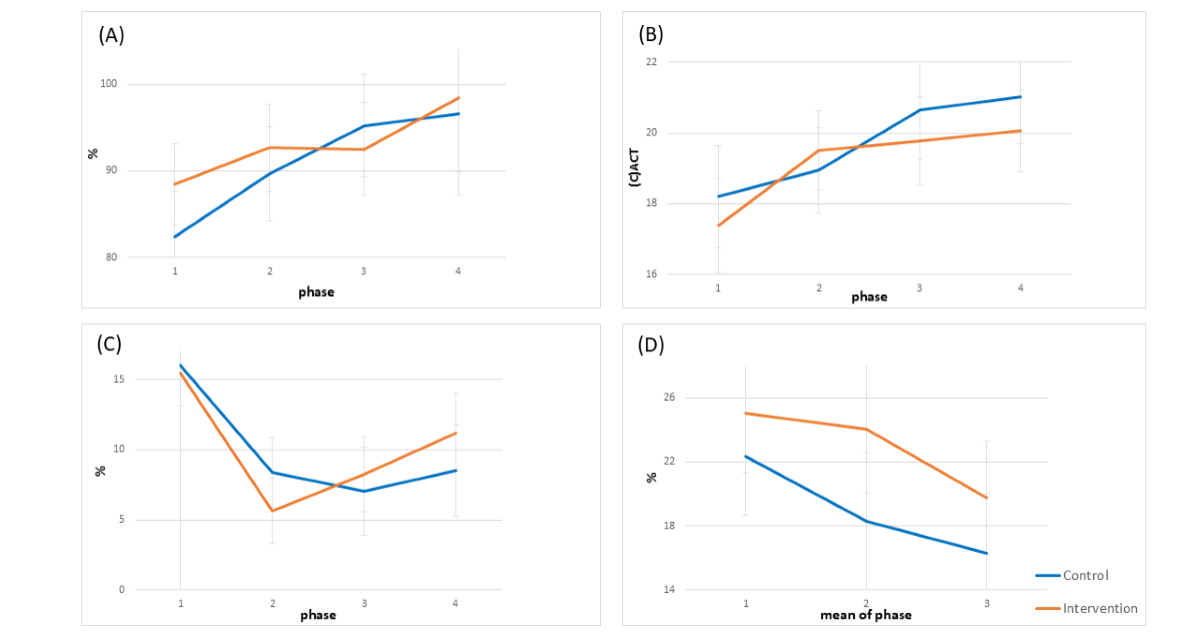
Time-by-group interaction of the mean change with reference to the baseline measure. (A) Predicted forced expiratory volume in 1 second percentage. (B) Asthma Control Test scores. (C) Lung function reversibility. (D) Lung function variability. The estimated marginal means of the mixed model with repeated measures are presented for each study visit measure per group: T0 (1) is the start of the study. T1 (2) is the start of phase 2, etc. In D, 1 is the mean of phase 1, 2 is the mean of phase 2, and 3 is the mean of phase 3.

In Figure 4A to 4D, the trends in the clinical parameters of asthma control over time for both groups are presented, with estimated means and standard errors.

### Predicted Forced Expiratory Volume per Second

Both groups exhibited a nonsignificant upward trend in predFEV_1_ percentage over time (*P*=.13 for the control group and *P*=.10 for the intervention group; Figure 4A). The between-group difference in the change in predFEV_1_ percentage over time did not reach statistical significance either (*P*=.36).

### Asthma Control Test

Both groups showed a significant increase in the C-ACT score over time (*P*=.01 for the control group and *P*=.04 for the intervention group), as shown in Figure 4B. The between-group difference in the absolute change in C-ACT score over time was not significant (*P*=.57).

### Lung Function Reversibility

The lung function reversibility for both groups showed a decrease over time (*P*=.02 for the control group and *P*=.12 for the intervention group). The between-group difference over time was not significant (*P*=.71; Figure 4C).

### Lung Function Variability

**Figure 5:**
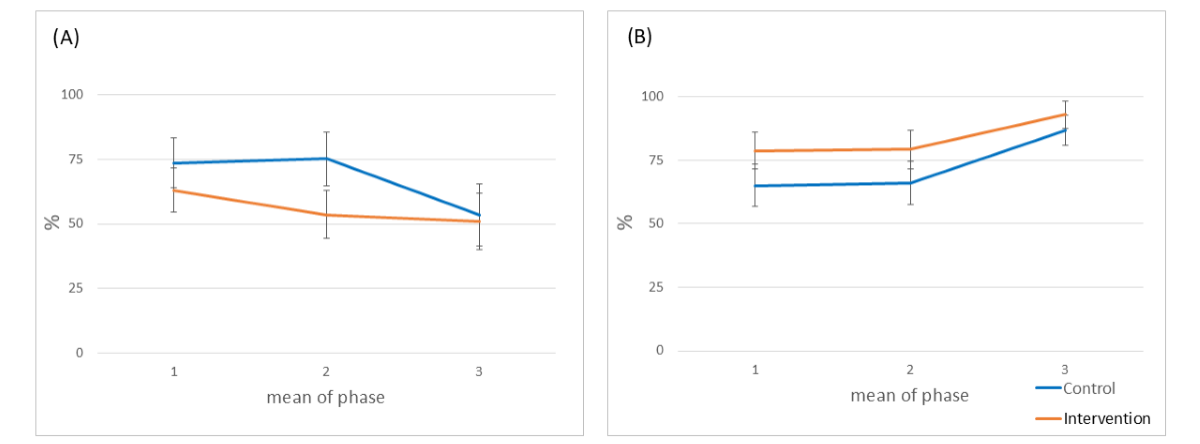
Time-by-group interaction of the mean change with reference to the baseline measure. (A) Adherence per phase. (B) Correct inhalations per phase. The estimated marginal means of the mixed model with repeated measures are presented for each phase per group: 1 is the mean of phase 1, 2 is the mean of phase 2, and 3 is the mean of phase 3.

A decrease in LFV for both groups was observed over time. The decrease was significant in the intervention group (*P*=.02) but not in the control group (*P*=.28; Figure 4D). The between-group difference in change in variability over time was not significant (*P*=.84; Figure 5D).

### Clinical Improvement

Multimedia Appendix 4 displays both the actual scores (performance) as well as the percentages of children fulfilling each of the 7 clinical criteria at the end of phase 2 in a table. Overall, no significant differences were observed between the control and intervention groups.

Criteria 4 and 5, which pertain to lung function reversibility, exhibited the highest fulfillment rates. A relative lung function reversibility decrease of ≥9% by the end of phase 2 was achieved in 73% of the participants in both the control (8/11) and intervention (11/15) groups. Furthermore, the criterion requiring a lung function reversibility of ≤12% following salbutamol administration at the end of phase 2 was met by 91% (10/11) of cases in the control group and 73% (11/15) in the intervention group. This criterion was already met at baseline, with 55% (6/11) and 53% (8/15) of the patients achieving this criterion during phase 2 (Multimedia Appendix 3).

In contrast, criteria related to LFV (criteria 6 and 7) demonstrated the lowest fulfillment rates in both groups, together with C-ACT score of ≥20 at the end of phase 2 (criterion 3). The criterion specifying an LFV of ≤15% throughout phase 2 was met by 27% (3/11) of participants in the control group versus 31% (4/13) in the intervention group. Furthermore, 36% (4/11) versus 46% (6/13) of the patients in the control and intervention groups, respectively, met the criterion for a relative LFV decrease of ≥10% during phase 2 compared with phase 1. Criterion 3 was met by 33% (4/12) and (5/15) of the participants in both groups, not including the children who had already fulfilled this criterion at baseline.

In total 87% (n/N) of the patients in the control group showed clinical improvement (ie, fulfilling one or more criteria at the end of phase 2) versus 78% (n/N) in the intervention group; this difference was not statistically significant (*P*=.52). The mean number of fulfilled criteria at the end of phase 2 in the control group was 2.33 (SD 1.54) versus 2.61 (SD 1.79) in the intervention group (*P*=.64)*.* Criteria that were already completed at baseline were not included in the total percentage of fulfillment per criterion.

### Therapy Adherence and Inhalation Technique

Figure 5A shows that, overall, both groups demonstrated a significant declining trend in adherence—significant in the control group (*P*=.03) and borderline significant in the intervention group (*P*=.05). The between-group difference over time was not significant (*P*=.14).

Both groups exhibited an upward trend in correct inhalations, with statistical significance in the control group (*P*=.005) and a similar borderline significant trend in the intervention group (*P*=.07). This overall improvement was particularly evident after phase 2. The between-group difference in change in correct inhalations over time was not significant (*P*=.70; Figure 5B).

The percentages of children who had good adherence (75%-125%) and a correct inhalation technique (≥75% correct inhalations) were calculated for each phase. Good adherence was observed in 57% (8/14) of participants in the control group and 50% (9/18) in the intervention group in phase 1. In phases 2 and 3, adherence remained relatively stable in the control group (54% and 50%, respectively). In the intervention group, it declined to 33% in phase 2 before returning to 50% in phase 3.

In phase 1, 43% (6/14) of participants in the control group and 72% (13/18) in the intervention group demonstrated good inhaler technique. In phase 2, these proportions increased to 62% (8/13) in the control group and remained relatively stable at 67% (10/15) in the intervention group. In phase 3, these increased to 90% (9/10) and 85% (11/13), respectively.

## Discussion

### Principal Findings

In this study, both the control and intervention groups demonstrated improvement over time in the clinical parameters—predFEV_1_ percentage, C-ACT, LFV, and lung function reversibility; however, there was no difference between the groups at the end of phase 2. In accordance with the study protocol, an interim analysis indicated futility, leading to early termination of the trial.

This is the first study that investigates the effects of smart immediate feedback and monitoring on objective and subjective clinical end points, providing valuable insights into patient and group characteristics and behavior in relation to pediatric asthma. Both the control and the intervention groups exhibited a high rate of clinical improvement at the end of phase 2 (87% vs 78% fulfilling one or more clinical criteria). Although we identified a clinical improvement for both groups at the end of phase 2, we did not observe improvement in adherence or inhalation technique during phase 2. Adherence declined during phase 2, which we believe reflects a component that is less related to skill acquisition and more to behavior. It is likely that adherence was overestimated at the start of the study—possibly due to participants initially demonstrating more favorable behavior than was truly representative—which then gradually decreased over time. Interestingly, a more notable improvement in inhalation technique emerged in phase 3 in the control group. This delayed effect may reflect the time required for a skill acquisition change to take hold, as is known from the stages of change in adherence and other health settings [34,35]. Initial exposure to feedback in phase 2 may have laid the foundation, known as the contemplation or preaction phase, with actual skill acquisition becoming evident only later. Participants may also have needed time to become familiar with the smart inhaler and its feedback mechanisms, leading to more effective use and readiness for the feedback over time [36]. However, this also raises the question of whether the feedback system was sufficiently intuitive for children. In our study, we used a system designed suitable for both adults and children, incorporating clear pictograms as part of the feedback. Nevertheless, a more child-specific and intuitive design might have facilitated faster adaptation. In addition, the auditory reminders used in the system may have elicited resistance, potentially leading participants to disable them. Although we were unable to monitor the exact frequency of reminder deactivation in our study, prior research by Knauer et al [37] in a different population suggests that social acceptance in this context is a critical factor in pediatric settings. Furthermore, the lack of an observed effect on inhalation technique and adherence suggests that other important factors may influence clinical asthma control beyond the parameters measured in this study, such as lifestyle, allergic exposure, or parental and environmental smoking habits. Standard care was not fully represented in our study, as both groups received a smart monitoring device—one group with active feedback and the control group with monitoring data transferred only to the researcher. Therefore, improvements observed in both groups may partly be explained by the monitoring principle itself, especially for adherence. This observation may also be partially attributable to the Hawthorne effect, in which improvements in observable behaviors, such as inhalation technique or adherence, are driven by the awareness of being observed [38]. However, these effects were not consistently observed in our study. It is possible that the Hawthorne effect instead manifested through other behavioral changes, such as lifestyle adjustments, which were not directly measured but may have contributed to the observed clinical improvements.

Given the episodic nature of asthma, patients experience fluctuations in symptoms throughout the year, with considerable interindividual variability. These dynamics underscore the potential value of continuous digital monitoring and feedback mechanisms. By capturing real-time changes in symptoms and behavior, such systems may support more personalized and responsive care. Moreover, a model based on objective monitoring to identify pitfalls could facilitate focused interventions and could potentially lead to more effective care. As part of a decision support tool, the smart inhaler must be able to work under various conditions, inconsistencies, and different patient needs [39]. More extensive monitoring may be used for a short period at the start of an eHealth strategy to identify personal cues that disrupt asthma control and to develop a personalized eHealth treatment strategy. Thereafter, monitoring intensity could be personalized to prevent alert fatigue, as observed in other decision support systems [40], while continuing the monitoring of the personal disrupting cues to allow timely short-term detection of asthma control deterioration. This resembles a just-in-time approach, in which the device is delivering when the need is high and receptiveness is likely [41,42], thereby empowering patients to take greater ownership of their condition [43,44].

A key strength of this study is the continuous use of the Respiro smart add-on device, which enabled objective assessment of both adherence and inhalation technique in an ambulatory, real-world setting. This approach enhanced the validity of our findings, ensuring that the results were representative of actual inhaler use in daily clinical practice. Another strength is the comprehensive approach to defining the clinical end points of asthma. The C-ACT questionnaire is the current validated gold standard in modern Dutch health care for assessing asthma control in children. However, the C-ACT comes with several drawbacks [45-47]. Asthma control in children fluctuates greatly, and the C-ACT questionnaire fails to account for this variability. The C-ACT score tends to underestimate asthma control, as defined by GINA [48,49]. However, to compensate for the drawbacks of the C-ACT questionnaire, other objective parameters, such as predFEV_1_, lung function reversibility after intake of medication, and LFV, were included.

### Limitations

Several limitations must be acknowledged. A substantial number of participants already met one or more of the criteria (eg, ACT score or lung function reversibility) at baseline, potentially limiting the scope for observable improvement. Additionally, for both groups, adherence to protocol, particularly concerning home measurements, was suboptimal, which may have affected the reliability of LFV as an outcome measure and should therefore be considered when interpreting its suitability as a parameter. The auditory warning signals provided by the smart inhaler were sometimes disabled by the participants in the feedback group, although no exact data on this were available. Although the intervention group initially demonstrated a significantly higher absolute FEV_1_ (in liters), no substantial effect was expected from this baseline difference, as predFEV_1_ percentage values were comparable between both groups. The study was limited by reduced statistical power, generalizability, and therefore by a limited ability to detect smaller effects. However, according to the stopping rule in the protocol, an effect size of less than 10% compared with the control group indicated futility, and therefore no substantial changes in outcomes were to be expected from including additional participants.

### Comparison With Prior Work and Future Perspectives

We believe that adding a smart monitoring feedback device (eg, Respiro) to the arsenal of asthma management provides a clearer view into patient behavior and presents an opportunity to enhance the efficiency and precision of asthma management strategies. Data from the observational phase of the IMAGINE study show that adherence and the inhalation technique are distinct and separate pitfalls in asthma care, each representing independent challenges [50]. In our study, adherence declined significantly in both groups, a finding similar to the Outcomes following Tailored Education and Retraining: Studying Performance and Adherence (OUTERSPACE) study, in which the intervention group showed a similar decline in adherence over time [51]. The authors explained this decline by the improvement in inhalation technique, which might reduce the need for strict adherence as the effectiveness of each inhalation increases. Furthermore, the absence of auditory reminders may also partly explain the decline in adherence observed in the OUTERSPACE study, a finding that could likewise apply to our study [51]. The INCA Sun study reported better adherence in the group treated with a digital inhaler compared with the control group. Similar to our study, the Inhaler Compliance Assessment (INCA) device measured data on treatment use and the inhaler technique in both study groups. However, feedback was not provided immediately, and therefore did not closely resemble our intervention [52]. In the Connected Electronic Inhalers Asthma Control Trial (CONNECT) 1 and 2 studies, the smart inhaler provided direct feedback on adherence and the inhalation technique through a patient-facing app. Both studies demonstrated improvements in clinical outcomes. Two key differences from our study are that the CONNECT trials included participants aged 13 years and older, and that their control groups received standard care without monitoring, whereas our control group was also monitored. Consequently, these differences in design and population may explain the contrasting findings regarding the impact of feedback on clinical outcomes [53,54]. Future research should focus on the optimal form of feedback, particularly adaptive and real-time interventions. As a first step, a prospective study could be designed in which a cohort of children with moderate to severe asthma is provided with a smart inhaler for an extended period [55]. At predefined moments—specifically when asthma control deteriorates or issues with adherence and the inhalation technique arise—additional tailored support could be offered to the intervention group, in contrast to standard nonadaptive care in the control group. Key questions include whether a just-in-time approach can improve outcomes, prevent exacerbations, and enhance self-management and user experience; an example of a protocol with this design is the Mobile Health for Kids With Asthma study [56]. Current clinical guidelines addressing pitfalls in asthma management may be too generic in mitigating issues of adherence and inhalation technique [55,57]. In contrast, objective monitoring and targeted attention to inhalation technique and adherence appear to contribute to more effective and efficient care. Such approaches not only improve outcomes but also enable a more personalized focus, fostering patient ownership in disease management [58].

Ultimately, this could pave the way for a study in which multicomponent adaptive interventions are delivered in a personalized manner, providing care for the right patient at the right time.

This creates a whole new opportunity to offer patient-tailored advice, enabling individuals to take responsible ownership of their condition while allowing health care providers to make optimal use of the just-in-time resources and opportunities for this disease with an episodic nature in the current health care landscape.

### Conclusions

In summary, this study investigated both objective and subjective clinical end points in the context of smart immediate feedback and monitoring for pediatric asthma management. Although the intervention did not lead to superior outcomes compared with the control group, the overall clinical improvements observed across both groups suggest that increased awareness and objective monitoring positively influence asthma control in children with uncontrolled asthma. The integration of smart monitoring feedback devices (eg, Respiro) offers valuable insights into patient behavior, thereby highlighting opportunities for more personalized and effective asthma management strategies. Based on our findings, we propose that future research should focus on refining assessment criteria and exploring the potential benefits of more targeted, personalized adaptive feedback interventions in asthma management.

## Appendix

Multimedia Appendix 1 CONSORT-eHEALTH checklist (V 1.6.1).

Multimedia Appendix 2 Equations 1 to 4.

Multimedia Appendix 3 Four domains divided into 7 clinical criteria for pediatric asthma control.

Multimedia Appendix 4 Performance and fulfillment per criterion. The performance on the 7 criteria for both groups is displayed as median and IQR, while the fulfillment of the criteria is displayed as percentage of subjects who fulfilled the criteria within the group. If criteria 3 and 5 were already fulfilled at baseline, they were not accounted for in the total percentage of fulfillment per criterion.

## Abbreviations

ACT: Asthma Control Test
C-ACT: Childhood Asthma Control Test
CONSORT-EHEALTH: Consolidated Standards of Reporting Trials of Electronic and Mobile Health Applications and Online Telehealth
FEV1: forced expiratory volume in 1 second
GINA: Global Initiative for Asthma
IMAGINE: Improving Adherence by Guiding Inhalation via Electronic Monitoring
LFV: lung function variability
predFEV1: predicted forced expiratory volume in 1 second

## Data Availability

The dataset generated or analyzed during this study is available from the corresponding author upon reasonable request.
