# Supplementary material for: Guided Inhalation via Electronic Monitoring in Children With Uncontrolled Asthma (the IMAGINE Study): Randomized Controlled Trial
Source: JMIR Pediatr Parent. 2025 Nov 14;8:e78526. doi: 10.2196/78526 (PMC12663702; doi:10.2196/78526)
Supplement: Multimedia Appendix 2 [file pediatrics_v8i1e78526_app2.docx]

**Multimedia appendix**

Equations

1. $Reversibility= \frac{FEV_{1}\left( L \right) post SABA -FEV_{1} \left( L \right) pre SABA}{FEV_{1} \left( L \right) pre SABA} \cdot100\%$
2. $Lung Function Variability=100\%-\frac{Minimum FEV_{1}\left( L \right)in phase x}{Maximum FEV_{1}\left( L \right) in phase x} *100\%$
3. *Adherence(%)* $=\frac{actual medication intake}{prescribed medication intake}$ x 100%
4. *Inhalations without critical errors(%)* $=\frac{correct number of inhalations (without critical error)}{total number of inhalations}$ x 100%
