# Supplementary material for: Guided Inhalation via Electronic Monitoring in Children With Uncontrolled Asthma (the IMAGINE Study): Randomized Controlled Trial
Source: JMIR Pediatr Parent. 2025 Nov 14;8:e78526. doi: 10.2196/78526 (PMC12663702; doi:10.2196/78526)

**Multimedia Appendix 2**

Four domains divided into seven clinical criteria for paediatric asthma control.


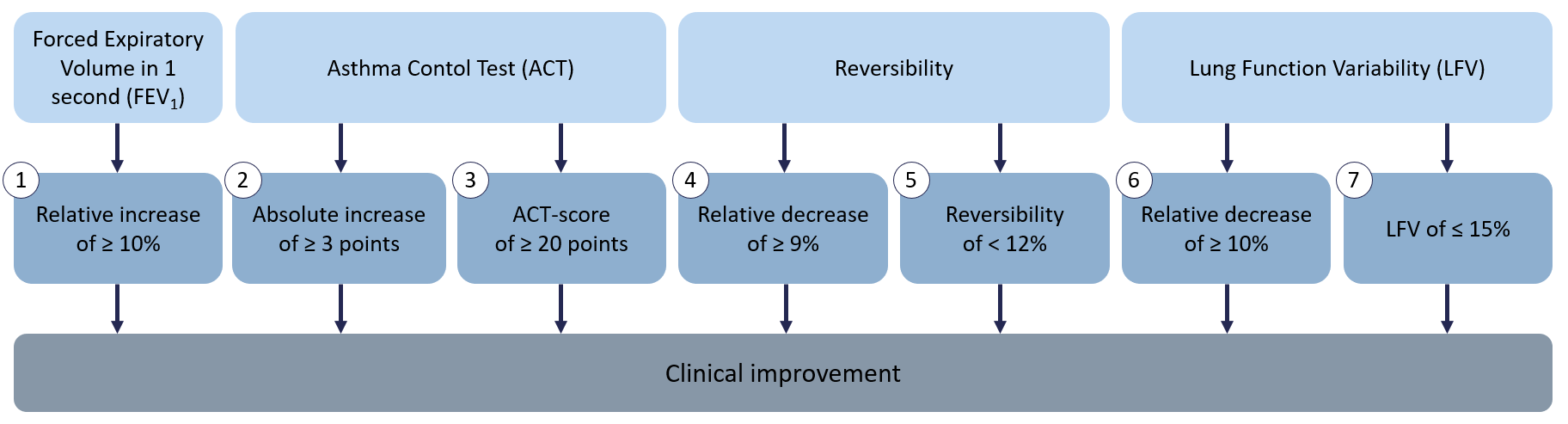

Supplement: Multimedia Appendix 3 [file pediatrics_v8i1e78526_app3.docx]
