# Supplementary material for: Guided Inhalation via Electronic Monitoring in Children With Uncontrolled Asthma (the IMAGINE Study): Randomized Controlled Trial
Source: JMIR Pediatr Parent. 2025 Nov 14;8:e78526. doi: 10.2196/78526 (PMC12663702; doi:10.2196/78526)
Supplement: Multimedia Appendix 4 [file pediatrics_v8i1e78526_app4.docx]

**Multimedia Appendix 3**

Performance and fulfilment per criterion.

| **Criterion** | | | **Control  group** | **Intervention group** | **Between-group analysis  (*P-*value)** |
| --- | --- | --- | --- | --- | --- |
| 1 | Relative FEV1 increase of ≥10% at the end of phase 2, compared to baseline | ∆FEV1 value(%) | 11.1 (1.3-22.8) | 8.2 (-7.8-13.5) | .24 |
|  |  | Fulfilment (%) | 55 (6/11) | 40 (6/15) | .69 |
| 2 | Absolute (c-) ACT score increase of ≥ 3 points at the end of phase 2, compared to baseline | ∆(c-)ACT score | 3 (1/5) | 3 (-1-5) | .77 |
|  |  | Fulfilment (%) | 55 (6/11) | 53 (8/15) | 1.00 |
| 3 | (c-) ACT score of ≥ 20 at the end of phase 2 | (c-)ACT score | 20 (17-22) | 21 (16-24) | .70 |
|  |  | Fulfilment (%) | 33 (4/12) | 33 (5/15) | 1.00 |
| 4 | Relative lung function reversibility decrease of ≥ 9% at the end of phase 2, compared to baseline | ∆LFR (%) | -39.2 (-75.1-29.4) | -61.2 (-90.4-29) | .51 |
|  |  | Fulfilment (%) | 73 (8/11) | 73 (11/15) | 1.00 |
| 5 | Lung function reversibility of ≤ 12% after administration of salbutamol at the end of phase 2 | LFR (%) | 6.6 (3.8-9.5) | 8.9 (2.1-13.4) | .70 |
|  |  | Fulfilment (%) | 55 (6/11) | 53 (8/15) | 1.00 |
| 6 | Relative LFV decrease of ≥ 10% during phase 2, compared to the LFV of phase 1 | ∆LFV (%) | -7.3 (-37-11) | -5.8 (-45.1-51.5) | .84 |
|  |  | Fulfilment (%) | 36 (4/11) | 46 (6/13) | .70 |
| 7 | LFV of ≤ 15% measured during the entire phase 2 | LFV (%) | 16.1 (14.5-22.4) | 18.6 (10.6-41.1) | .40 |
|  |  | Fulfilment (%) | 27 (3/11) | 31 (4/13) | 1.00 |

^The performance on the seven criteria for both groups is displayed as median and interquartile ranges, the fulfilment of the criteria is displayed as percentage of subjects who fulfilled the criteria within the group. If criteria 3 and 5 were already fulfilled at baseline, they were not accounted for in the total percentage of fulfilment per criterion.^
